# Systemic Mastocytosis: A Rare Case of Increased Liver Stiffness

**DOI:** 10.1155/2012/728172

**Published:** 2012-10-11

**Authors:** Stefanie Adolf, Gunda Millonig, Helmut Karl Seitz, Andreas Reiter, Peter Schirmacher, Thomas Longerich, Sebastian Mueller

**Affiliations:** ^1^Department of Medicine, Salem Medical Center and Alcohol Research Center, University of Heidelberg, Zeppelinstraße 11-33, 69121 Heidelberg, Germany; ^2^Department of Medicine III, Mannheim Hospital, University of Heidelberg, Wiesbadener Straße 7-11, 68305 Mannheim, Germany; ^3^Institute of Pathology, University of Heidelberg, Im Neuenheimer Feld 220/221, 69120 Heidelberg, Germany

## Abstract

Assessment of liver stiffness (LS) by transient elastography (Fibroscan) has significantly improved the noninvasive diagnosis of liver fibrosis. We here report on a 55-year-old patient with drastically increased LS due to previously unknown systemic mastocytosis. The patient initially presented with increased weight loss, nocturnal pruritus, increased transaminases, bilirubinemia, and thrombocytopenia. Abdominal ultrasound showed ascites, hepatomegaly, and splenomegaly. In addition, LS was 75 kPa (IQR 0 kPa) clearly exceeding the cut-off value for F4 cirrhosis of 12.5 kPa. However, histological analysis of the liver specimen indicated liver involvement by systemic mastocytosis and excluded liver cirrhosis. An additional CT scan detected disseminated bone lesions. After three months of treatment with Midostaurin, LS slightly decreased down to 31.9 kPa (IQR 8.3 kPa). This case illustrates that diffused sinusoidal neoplastic infiltrates are a pitfall in the non-invasive diagnosis of liver cirrhosis. In conclusion, refined clinical algorithms for increased LS should also include mastocytosis in addition to inflammation, congestion, and biliary obstruction.

## 1. Introduction

In the last six years, transient elastography [TE, Fibroscan] has become an established key tool for the rapid and non-invasive assessment of liver fibrosis and cirrhosis [1]. TE determines liver stiffness (LS) with high reproducibility in about 95% of all patients and LS has been shown to be in excellent agreement with the degree of fibrosis stage in patients with various liver diseases [2–5]. On the basis of these studies, a cut-off value of above 12.5 kPa has been elaborated for the discrimination of liver cirrhosis (F4) while LS values below 6 kPa are considered as normal [6, 7]. Liver biopsy which is the gold standard for assessing hepatic fibrosis or cirrhosis is an invasive procedure, with rare but potentially severe complications. In addition, the accuracy of liver biopsy in assessing fibrosis has limitations because of well-known sampling errors and interobserver variability [8–12]. Nevertheless, liver biopsy and measurement of LS should be regarded as synergistic diagnostic approaches. Thus, while the sampling error of TE is significantly less with regard to fibrosis assessment as compared to biopsy, histology provides many valuable diagnostic information. In addition, factors have been identified that increase LS irrespective of fibrosis. Such factors include hepatic congestion [13], inflammation [14, 15], or cholestasis [16]. To avoid potential misinterpretations of increased LS, the precise knowledge of these factors has an important impact on the usage of TE for fibrosis assessment. Recently, new refined algorithms have been developed to increase the diagnostic accuracy [7, 17] of LS that include a timely abdominal ultrasound and laboratory tests. Here we describe a case of hepatic involvement in systemic mastocytosis with drastically elevated LS in the absence of cirrhosis. Thus, a further clinical entity is added to the differential diagnosis of increased LS underscoring the necessity of accurate disease typing for LS assessment. 

## 2. Case Presentation 

A 55-year-old man was admitted to our hospital with weight loss of 20 kg in 6 months, fatigue, and increasing nocturnal pruritus mainly in lower extremities. The remaining patient's history was uneventful. The physical examination was normal except for slightly enlarged painless submandibular lymph nodes. Chest X-ray and electrocardiogram were normal. Initial laboratory tests showed anemia (Hb 9.6 g/dl) and significant thrombocytopenia (65/nl). White blood count was elevated (15.8/nl) as was the C-reactive protein with 21.7 mg/l (normal <0.5). Liver enzymes were elevated (GOT 65 U/l, GPT 74 U/l, GGT 329, AP 830 U/l, bilirubin 2.4 mg/dl) while synthesis parameters such as albumin and INR were normal. Serum ferritin was also elevated (581 ng/ml).

 Abdominal ultrasound showed ascites and an enlarged liver (craniocaudal diameter of 19 cm) and spleen (16.3 cm). Liver echogenicity was homogenous and there were no classical signs of liver cirrhosis detectable such as nodular aspect of the liver surface or collaterals such as a revascularized umbilical vein. LS assessed by Fibroscan (XL probe) was drastically increased with 75 kPa (IQR 0 kPa, success rate 100%). This value represents the upper detection limit of the Fibroscan device and exceeds by far the cut-off value of cirrhosis (F4; 12.5 kPa). An additional CT scan revealed disseminated bone metastasis and was suspicious of peritoneal carcinomatosis. Endoscopy of the upper and lower gastrointestinal tract was normal.

**Figure 1:**
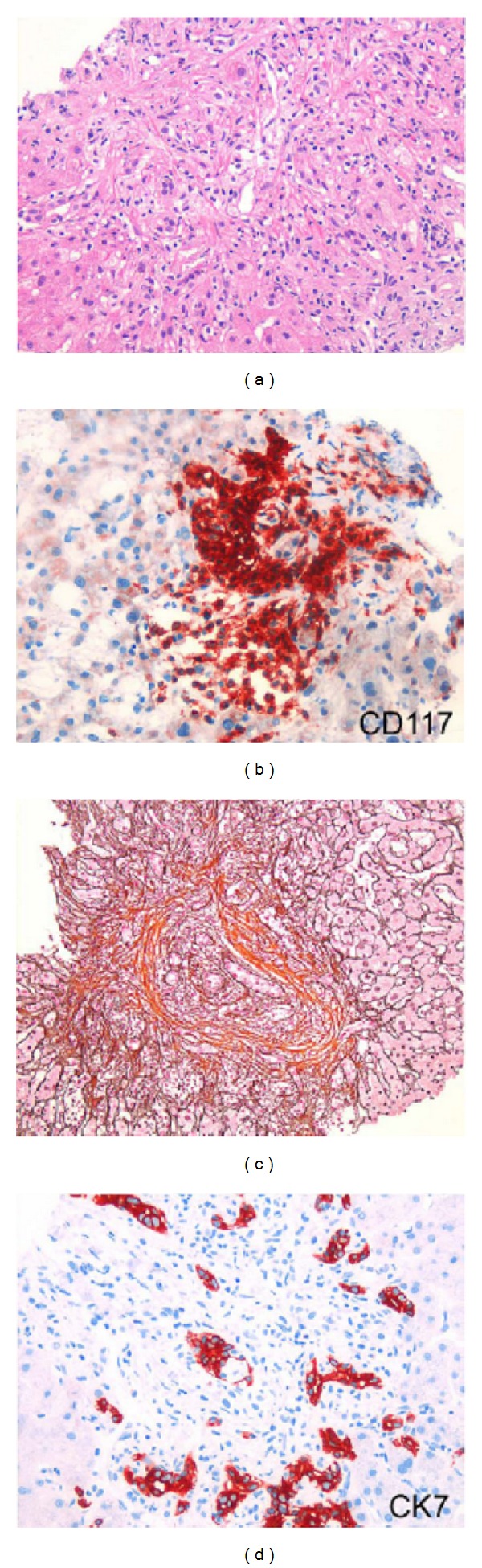
Liver biopsy of the patient with drastically increased liver stiffness due to mast cell infiltrates. (a) Infiltration of portal tract and liver sinusoids with neoplastic mast cell like cells (HE). (b) CD117 immunostaining shows nodular infiltration with mast cells. (c) Portal, periportal, and perisinusoidal fibrosis (modified Gomori stain. 200×). (d) CK7 immunostain identifies neoplastic mast cells that infiltrate the portal bile ducts and ductular structures 200×.

A liver biopsy was performed that revealed a mild portal fibrosis but excluded liver cirrhosis (Figure 1). In contrast, there were portal, sinusoidal, and micronodular infiltrates of spindle cells with round to oval nuclei with dense chromatin and moderately developed pale cytoplasm. These cells were positive for CD117 (c-kit) and CD68 and negative for CD1a by immunohistochemistry and were thus identified as mast cells. 

**Figure 2:**
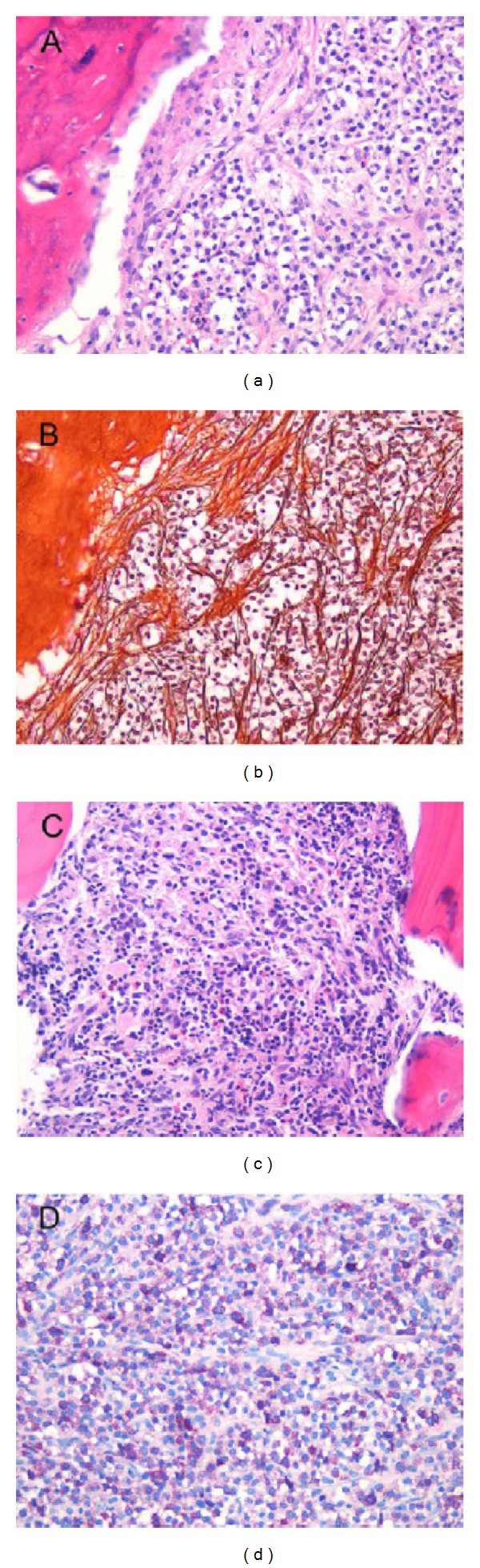
Bone marrow specimen of the patient with systemic mastocytosis. (a) Diffuse infiltration of the bone marrow with neoplastic mast cells and massive displacement of hematopoetic cells (HE). (b) Induction of myelofibrosis by neoplastic cells (modified Gomori stain). (c) Residual hematopoiesis besides neoplastic infiltrates (HE). (d) Giemsa staining shows cytoplasmatic granules of neoplastic mast cells 200×.

A cutaneous manifestation of the mastocytosis was evident by reddening of body and lower extremities and red papules pretibially and was confirmed by a skin biopsy. During staging a bone marrow biopsy was taken which demonstrated an extensive bone marrow infiltration by atypical mast cells and a significantly reduced haematopoiesis (Figure 2). Genetic analysis of the bone marrow allowed the identification of a point mutation (D816V) within the kit gene (c-Kit) which is known to be responsible for hyperactivation of the growth receptor Kit (tyrosine kinase) on the mast cell surface [18]. Thus, the diagnosis of systemic mastocytosis was established in this patient. Accordingly, serum tryptase levels were found to be elevated with 200 ng/l (normal 1–11.3 ng/ml).

The excessive infiltration of the bone marrow and other extracutaneous organs with mast cells suggested that the patient suffered from aggressive systemic mastocytosis. This was confirmed by the presence of several additional key criteria for the aggressive form (so-called c-findings) such as cytopenia, enlarged liver and spleen, ascites, malabsorption, and weight loss. Consequently, immediate treatment with a tyrosine kinase inhibitor was initiated. Dasatinib was used since Kit-D816V positive patients are known to be resistant against Imatinib. In addition, the patients pruritus was treated with H-1 blockers. Proton pump inhibitors were administered to prevent gastric ulcer development. Since the patient did not respond to Dasatinib within 6 weeks and diarrhea deteriorated, he was enrolled in the PKC412 study with Midostaurin. Restaging after 6 weeks also showed progressive disease including a further enlargement of spleen, liver as well as cervical, axillar, and inguinal lymph nodes, an increase of tryptase levels to 410 *μ*g/l, an increasing diffuse sclerosis of the skeleton, and an increased bone density. At the moment, the patient has slightly improved after three months of treatment with Midostaurin and liver stiffness decreased down to 31.9 kPa (IQR 8.3 kPa, success rate 100%). 

## 3. Discussion

### 3.1. Systemic Mastocytosis

Mastocytosis is defined as clonal expansion and accumulation of mast cells in at least one organ [19]. Incidence is low with five cases per 1 million people; most of the cases occur in children. According to the WHO, major manifestations include cutaneous or systemic forms [20]. The D816V point mutation within the tyrosine kinase Kit (*c-Kit*) that is detected in 80% of cases is considered a driver mutation causing the permanent receptor activation and consequent proliferation, and thus neoplastic expansion of the mutated mast cell clone. The activation of several additional oncogenes and other mutations probably contributes to the development of the more aggressive form present in our patient [21]. General symptoms such as hypotension, flush, and dyspnea are directly caused by mast cell activation while local symptoms are due to organ infiltration. Bone marrow involvement is present in most patients (90%) followed by involvement of gastrointestinal tract, liver, spleen, and lymph nodes. Gastrointestinal manifestations are unspecific and include abdominal pain, nausea, vomiting, and diarrhea [22, 23]. For complete staging of systemic mastocytosis, a bone marrow biopsy is required. At present, tyrosine kinase inhibitors are the major treatment strategy. Imatinib is rarely used since 80% of patients have the D816V genotype and are therefore resistant to this drug. Dasatinib is more potent and well tolerated [24]. Novel drugs include Nilotinib (BCR-ABL tyrosine kinase inhibitor) and Midostaurin [25]. Midostaurin is a potent inhibitor of the tyrosine kinase FTL3 (Fetal-Liver-Tyrosine-Kinase 3), a receptor of the hematopoetic precursor cell. A recent phase II study on 20 patients with refractory AML or high-grade myelodysplastic syndrome showed a significant reduction of peripheral blasts [26]. At the moment, Midostaurin is studied in phase III for the treatment of acute myeloid leukemia and aggressive form of systemic mastocytosis [27].

### 3.2. Increased Liver Stiffness by Systemic Mastocytosis

The present case is remarkable for two reasons. First, it is the first description of a drastically increased LS in a patient with hepatic involvement by systemic mastocytosis. Second, it adds a further if not rare clinical entity to the differential diagnoses of increased LS. Although LS is now well established as excellent non-invasive parameter for the assessment of liver cirrhosis, other clinical conditions such as hepatic congestion [13], inflammation [14, 15], or cholestasis [16] are known to drastically increase LS independent of fibrosis stage. In addition, deposition of hepatic amyloid was shown to increase LS in the absence of cirrhosis [28, 29]. 

**Figure 3:**
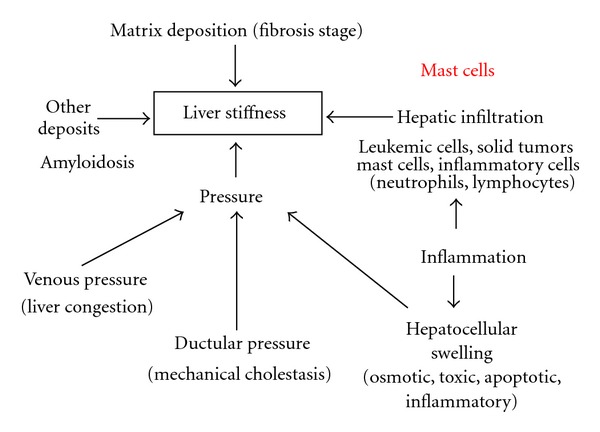
Factors that affect liver stiffness including mast cell infiltration.

We have recently suggested that these LS interfering conditions can be separated in two major categories [7, 17]: either they are caused by deposition of matrix such as collagen during fibrogenesis or they are associated with an increase in intrahepatic pressure such as in mechanic cholestasis or liver congestion (Figure 3). Inflammation-dependent increase of LS is also predominantly pressure driven since it is accompanied by infiltration with inflammatory cells, increase of interstitial fluid, and increase of cellular size (ballooning). The major goal for an optimal diagnostic interpretation of LS, therefore, is exact disease typing to clearly discriminate in a patient between matrix- and pressure-associated conditions. For these reasons, we consider abdominal ultrasound and laboratory tests mandatory for the correct interpretation of LS by TE [7]. Ultrasound imaging is a very robust and rapid way to exclude manifest cholestasis, nodular liver masses, or congestion. 

Our present case of systemic mastocytosis in which diagnosis was primarily established by liver biopsy fits within this concept. Under these conditions, the liver was massively infiltrated by mast cells which, in turn, caused an increase of liver volume and LS, consequently. Since the patient also showed increased transaminases, bilirubin levels, thrombocytopenia, and ascites, he could have been easily mistaken for having cirrhosis by superficial screening although some of his complaints were atypical for liver cirrhosis such as the rapid weight loss and nocturnal pruritus. From the basic exams, normal liver synthesis parameters and the absence of classical sonographic signs of cirrhosis are highly indicative that liver cirrhosis might not be the correct diagnosis. The increased LS seems to be predominantly due to mast cell infiltration since transaminase levels were only mildly increased and below 100 U/l. Moreover, during treatment, LS decreased by 50% in the absence of increased transaminases. False positive elevation of LS due to ascites can also be ruled out although ascites had been previously regarded as an exclusion criterion for transient elastography. Recently, we could demonstrated that (a) TE can be performed in the presence of ascites using the XL or sometimes even the M probe (b) consequent increased intra-abdominal pressure does not affect LS and (c) a normal LS allows to exclude hepatic causes of ascites [30].

**Figure 4:**
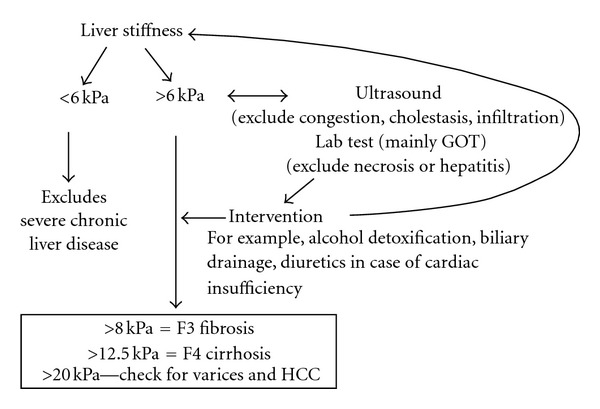
Present clinical algorithms for the interpretation of increased liver stiffness.

Figure 4 shows an updated diagnostic algorithms for the interpretation of LS. At present, with the use of the available probes (S, M, and XL probe), 95% of all patients can be measured by TE. While a normal LS rules out any severe chronic liver disease [7], abdominal ultrasound and lab tests should be performed in the case of increased LS to exclude congestion, cholestasis, or, as shown here, infiltration with mast cells. In cases of elevated transaminases higher than 100 U/l, diagnosis of cirrhosis is also uncertain unless LS exceeds 30 kPa. To discriminate between cirrhosis and these interfering factors, interventions are necessary such as antidiuretic therapy, decompression of the bile duct, for example, by biliary drainage, or treatment of the underlying hepatitis, for example, alcohol detoxification [13, 16, 17]. These interventions will eventually remove the “pressure associated component” of increased LS. We recently demonstrated that reassessment of LS after such interventions correlated much better with fibrosis stage [17]. Our case also shows that LS may actually prompt in some cases to a liver biopsy. Previously, there had been the fear that LS measurements will spare most patients from liver biopsies [5, 31] and indeed there are certainly situations now where liver biopsies can be omitted or postponed. However, as shown in our case, increased LS in the context of conflicting clinical findings is indicative that further histological evaluation may be necessary to finally settle the diagnosis.

In our opinion, the case further demonstrates that increased LS cannot be interpreted as liver cirrhosis automatically. Instead, exact disease typing supported by additional information such as ultrasound imaging, laboratory tests, and other clinical findings is critical for the correct interpretation of LS. In some cases, the pathologically elevated LS may even prompt the patient to further histological analysis. Therefore, we add mast cell infiltration as additional LS increasing condition. 

## Abbreviations

LS: Liver stiffness
TE: Transient elastography.
